# 

**Published:** 1881-08

**Authors:** 


HERMAN MYNTER, M. D.

### CONTENTS:

| | PAGE. |
|---|---|
| ORIGINAL COMMUNICATIONS: | |
| Some Practical Hints on the Fitting of Spectacles. By Frank W. Abbott, M. D., | 1 |
| CLINICAL REPORTS: | |
| Puerperal Convulsions—Induced Labor—Death of the Mother. Reported by H. R. Hopkins, M. D., | 11 |
| TRANSLATIONS: | |
| The Medico-Legal Import of the Opium Habit—By Ed. Levinstein. Translated from the German by Herman Mynter, M. D., | 14 |
| SELECTIONS: | |
| Coition in Pregnancy. By Theophilus Parvin, M. D., | 19 |
| The Tongue. By J. Milner Fothergill, M.D. | 24 |
| The Alkalinity of the Blood, | 31 |
| Relation of Foul Air to Consumption, | 32 |
| Hygienic Value of Mirth, | 33 |
| How Milk Should be Taken, | 34 |
| Mycosis of the Trachea, | 35 |
| Case of Remarkable Low Temperature, | 35 |
| Ovariotomy and Parotitis, | 36 |
| Artificial Nourishment of Infants, | 36 |
| Successful Case of Resection of Cancerous Stomach, | 37 |
| Corrosive Sublimate in Dysentery, | 38 |
| Treatment of Frequently Recurring "Erysipelas" of the Face, | 38 |
| Foreign Substance Introduced into the Brain with Impunity, | 38 |
| Psychical Impotence, | 39 |
| Three Views of a Consultation, | 40 |
| EDITORIAL: | |
| Volume XXI, | 40 |
| Buffalo State Insane Asylum. Resignation of Prof. James P. White, | 41 |
| A Word to Book Agents, | 43 |
| Alumni Association of the Albany Medical College, | 45 |
| REVIEWS, | 47 |

